# Supplementary material for: SARS-CoV-2 Infection-Induced Alterations in ADAR Editing Patterns Differ Between Patients Who Developed Critical Compared to Non-Critical COVID-19
Source: Int J Mol Sci. 2026 Jul 29;27(15):6809. doi: 10.3390/ijms27156809 (PMC13466449; doi:10.3390/ijms27156809)
Supplement: Supplementary file 1 [file ijms-27-06809-s001.zip › ijms-4385169-supplementary/SupplementaryMaterialsLegends.pdf]

Supplementary Materials for: Aiswarya Mukundan Nair, Helen Piontkivska, "SARS-CoV-2 infection induced alterations in ADAR editing patterns differ between patients who developed critical compared to non-critical COVID-19"

Supplementary materials and code are shared at https://github.com/RNAdetective/Editing_in_critical-non-critical-COVID-19

List of supplementary tables and figures:

(see Supplementary Figures below)

Supplemental Figure S1. PCA plot of top 500 differentially expressed genes. PC1 clearly separated critical from non-critical patients.
Supplemental Figure S2. Difference in ADAR editing between critical and non-critical patients (A) Average number of ADAR edits between critical and non-critical patients. (B) Region wise distribution of uniquely edited sites within non-critical patients. inset plots show different types of substitutions within exonic regions. (C) Region wise distribution of uniquely edited sites within critical patients. inset plots show different types of substitutions within exonic regions. (D) Screenshot of interactive 3D viewer showing interatomic interactions of the wildtype residue (S) at position 164 in exon 3 in HLA-DRB5 gene carrying non-synonymous substitution in non-critical patients. (E) Screenshot of interactive 3D viewer showing interatomic interactions of the wildtype residue (H) at position 292 in exon 7 in DHRSX gene carrying non-synonymous substitution in critical patients.
Supplemental Figure S3. Genomic distribution of differentially edited sites. Highest number of differentially edited sites mapped to introns followed by 3'UTR, exonic region also contains a missense edit.

(see Supplementary Tables as separate XLSX files)

Supp_Table_S1.xlsx

Supplemental_Table_S1A: DESeq2 differential gene expression analysis results comparing Critical COVID-19 samples to Non-critical COVID-19 samples, filtered for log2Fold Change >= |0.58| (Fold Change > 1.5) and an adjusted P value < 0.05

Supplemental_Table_S1B: DESeq2 differential gene expression analysis, genes upregulated in Critical COVID-19 samples compared to Non-critical COVID-19 samples, filtered for log2Fold Change >= 0.58 (Fold Change > 1.5) and an adjusted P value < 0.05

Supplemental_Table_S1C: DESeq2 differential gene expression analysis, genes downregulated in Critical COVID-19 samples compared to Non-critical COVID-19 samples, filtered for log2Fold Change =< -0.58 (Fold Change > 1.5) and an adjusted P value < 0.05

Supplemental_Table_S1D: Gene Set Enrichment Analysis of Reactome pathways enriched among differentially expressed genes ordered by Nominal Enrichment Score (NES) and p-adj < 0.05

Supplemental_Table_S1E: Gene Set Enrichment Analysis of GO-BP enriched among differentially expressed genes, ordered by Nominal Enrichment Score (NES) and p-adj < 0.05

Supplemental_Table_S1F: DESeq2 differential gene expression analysis results comparing Critical COVID-19 samples to Non-critical COVID-19 samples before applying filters of log2Fold Change >= |0.58| (Fold Change > 1.5) and an adjusted P value < 0.05

Supplemental_Table_S1G: Transcript level ADAR expression as DESeq2 normalized counts. Isoform transcript Ids were retrieved from Ensembl

Supplemental_Table_S1H: Transcript level ADAR expression data from DESeq2

Supp_Table_S2.xlsx

Supplemental_Table_S2A: Sample wise total number of different substitution types, including A-to-G and T-to-C substitutions

Supplemental_Table_S2B: Average region-wise number of ADAR editing sites within each patient group

Supplemental_Table_S2C: Editing sites and corresponding genes unique edited in patients with non-critical COVID-19

Supplemental_Table_S2D: Editing sites and corresponding genes unique edited in patients with critical COVID-19

Supplemental_Table_S2E: GO-BP overrepresentation analysis of uniquely edited genes

Supplemental_Table_S2F: Reactome pathway overrepresentation analysis of uniquely edited genes

Supp_Table_S3.xlsx

Supplemental_Table_S3A: List of identified high confidence sites annotated with snpEff

Supplemental_Table_S3B: High confidence sites present in REDI portal

Supplemental_Table_S3C: High confidence sites not-present in REDI portal/ Novel sites

Supplemental_Table_S3D: High confidence ADAR sites differentially edited between critical compared to non-critical patients

Supplemental_Table_S3E: Differentially edited ADAR edited sites within differentially expressed genes

Supplemental_Table_S3F: GSEA results of differentially edited sites using Reactome

Supp_Table_S4.xlsx

Supplemental_Table_S4: Features/edited coordinates with AUC>0.75

Supp_Table_S5.xlsx

Supplemental_Table_S5: Metrics of analyzed RNA-seq data from Bioproject PRJNA722046; GSE172114 (Carapito et al.,2022).

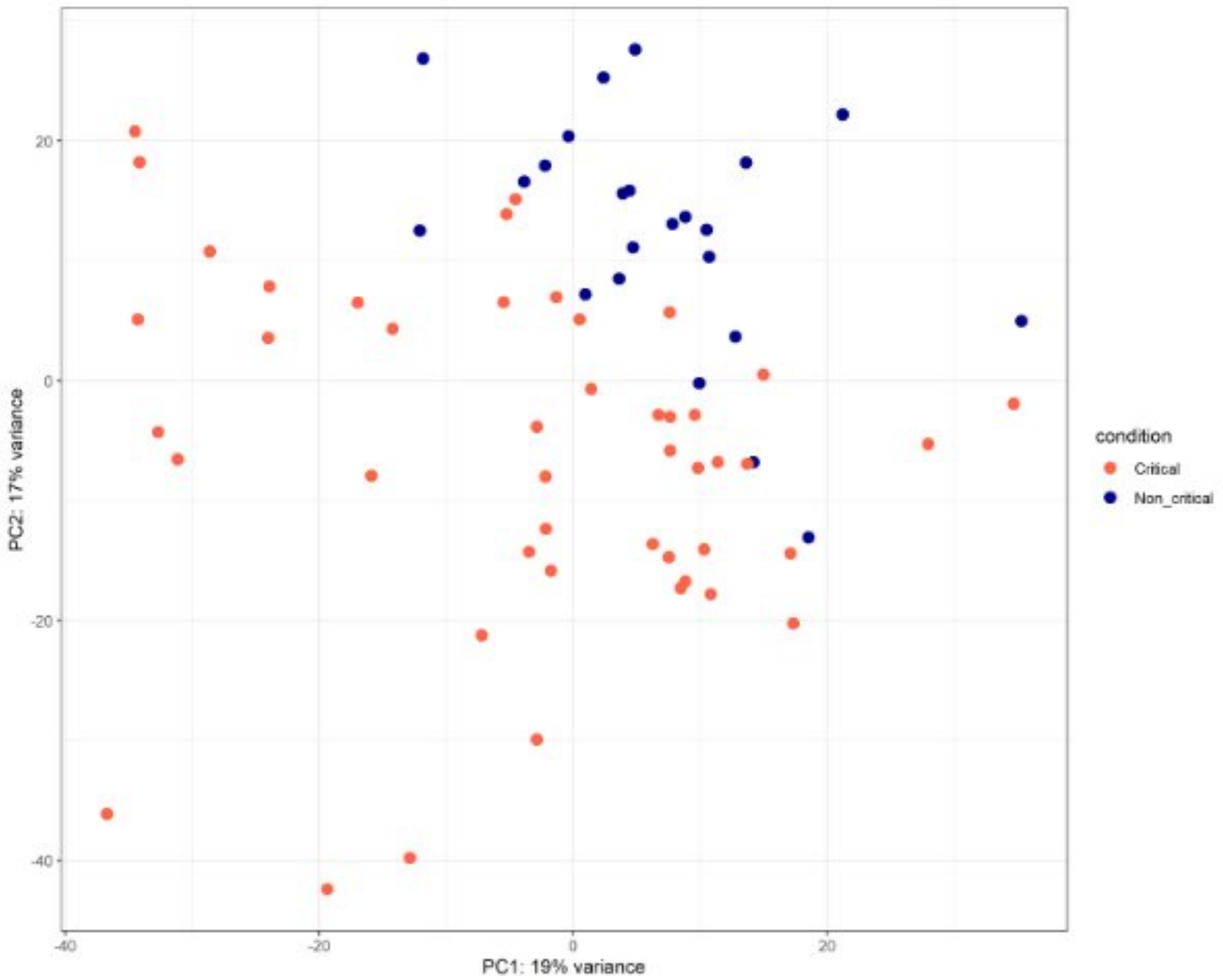

Supplemental Figure S1. PCA plot of top 500 differentially expressed genes. PC1 clearly separated critical from non-critical patients.

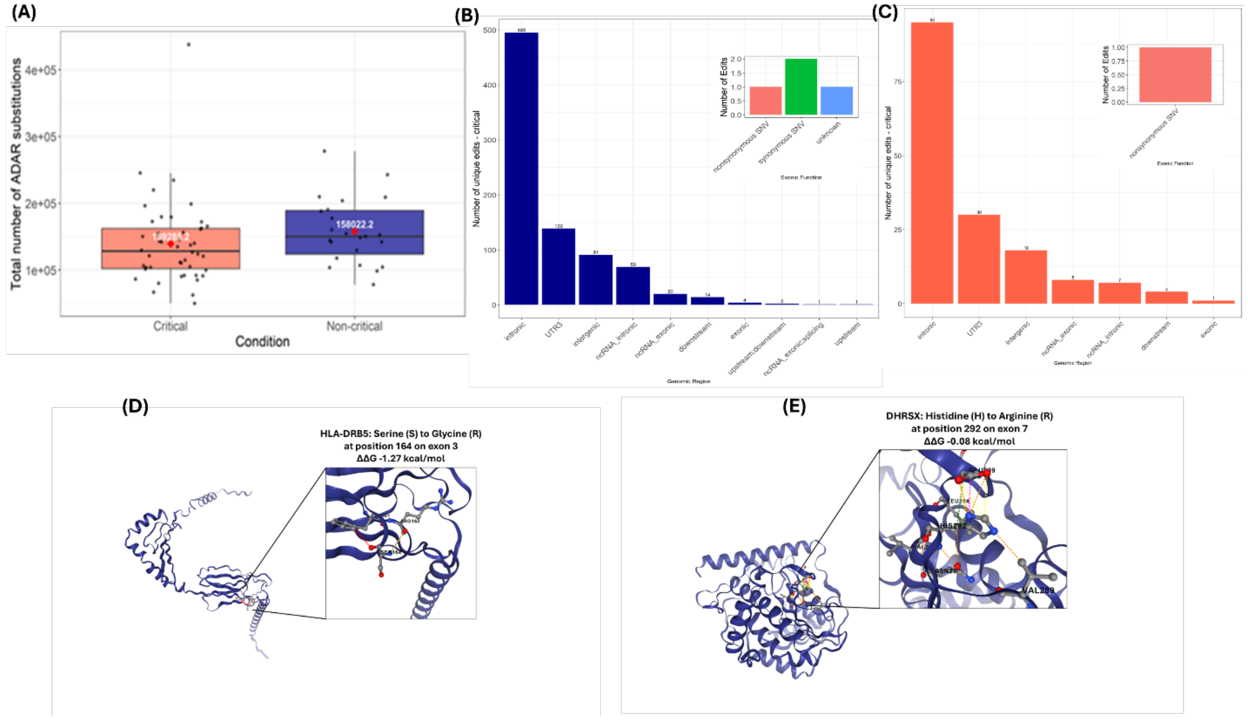

Supplemental Figure S2. Difference in ADAR editing between critical and non-critical patients (A) Average number of ADAR edits between critical and non-critical patients. (B) Region wise distribution of uniquely edited sites within non-critical patients. inset plots show different types of substitutions within exonic regions. (C) Region wise distribution of uniquely edited sites within critical patients. inset plots show different types of substitutions within exonic regions. (D) Screenshot of interactive 3D viewer showing interatomic interactions of the wildtype residue (S) at position 164 in exon 3 in HLA-DRB5 gene carrying non-synonymous substitution in non-critical patients. (E) Screenshot of interactive 3D viewer showing interatomic interactions of the wildtype residue (H) at position 292 in exon 7 in DHRSX gene carrying non-synonymous substitution in critical patients.

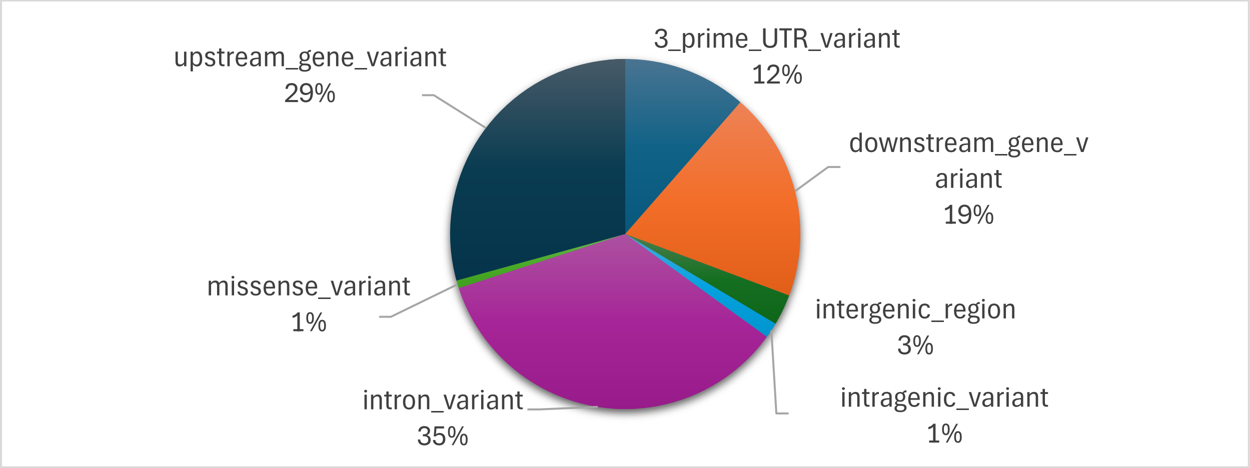

Supplemental Figure S3. Genomic distribution of differentially edited sites. Highest number of differentially edited sites mapped to introns followed by 3'UTR, exonic region also contains a missense edit.
